# Seroprevalence and Risk Factors for *Theileria equi* Infection in Equines from Khyber Pakhtunkhwa Province, Pakistan

**Published:** 2017

**Authors:** Muhammad Jamal Khan AFRIDI, Abdul Hafeez MIAN, Muhammad SAQIB, Ghazanfar ABBAS, Javid ALI, Muhammad Khalid MANSOOR, Awais ur Rahman SIAL, Imaad RASHEED, Muhammad Hammad HUSSAIN

**Affiliations:** 1. Faculty of Veterinary Sciences, University of Agriculture, Faisalabad, Pakistan; 2. Dept. of Parasitology, University of Veterinary and Animal Sciences, Lahore, Pakistan; 3. Faculty of Animal Husbandry and Veterinary Sciences, University of Agriculture, Peshawar, Pakistan; 4. Animal Health Research Center, Ministry of Agriculture and Fisheries, Muscat, Oman; 5. Dept. of Clinical Studies, PMAS Arid Agriculture University, Rawalpindi, Pakistan

**Keywords:** Seroprevalence, Piroplasmosis, *Theileria equi*, Donkey, Horse, cELISA, Pakistan

## Abstract

**Background:**

*Theileria equi* is a tick borne protozoan parasite which causes piroplasmosis among equines worldwide. The present study was aimed to determine seroprevalence of *T. equi* in donkeys, horses, and mules from two equine populated districts (Peshawar and Charsadda) of Khyber Pakhtunkhwa (KPK), Pakistan.

**Methods:**

A total of 393 equine (195 horses, 194 donkeys and 4 mules) serum samples were collected from five and four randomly selected localities in Charsadda (n = 193) and Peshawar (n = 200), respectively. The presence of antibodies to *T. equi* was determined using a commercially available competitive enzyme-linked immunosorbent assay.

**Results:**

An overall seroprevalence of 38.2% (n=150) was observed among all the tested animals suggesting a higher seropositivity among equids belonging to Charsada (50.3%) as compared to Peshawar (27.5%). Binary logistic regression analysis revealed that being a donkey (OR 2.94), having tick infestation (OR 4.32), history of voiding red (i.e., blood containing) urine (OR 3.97) and anemia (OR 2.1) were the factors significantly associated with the seroprevalence of *T. equi.* For animals with higher anti-*T. equi* antibody titers, a strong association of seroprevalence for *T. equi* was recorded with species, age, sex, tick infestation, anemia and history of hematuria.

**Conclusion:**

The present study indicates a high level of exposure of working equids to *T. equi* in KPK region, Pakistan. Future studies should focus on tick vector identification and other factors responsible for spread of the disease.

## Introduction

Equine piroplasmosis (EP) is caused by two protozoan haemoparasites, *Babesia caballi* and/or *Theileria equi* (1,2) and is mainly transmitted by species of ixodid ticks such as *Amblyomma*, *Dermacentor*, *Hyalomma* and *Rhipicephalus* (3,4). However, iatrogenic transmission (blood-contaminated syringes, needles, surgical instruments and by transfusion of infected blood and blood products) is not common in developing countries. EP is endemic in tropical and subtropical regions of the world, including Asia, Africa, parts of Southern Europe and Russia, south and central Americas and certain regions of the USA (5).

*Theileria equi* infected equids usually exhibit a severe and acute form of EP; whereas, *B. caballi* infection usually follows a chronic course (3, 6–8) and a number of factors, including infecting dose, genetics of the infecting parasites and immune status of the host etc. can affect the severity of the disease (3). Clinically, EP is characterized by pyrexia, anorexia, depression, jaundice, hemoglobinuria, bilirubinuria, regenerative haemolytic anaemia and even mortality (3, 9, 10). However, sub-clinical infections are commonplace, particularly in endemic regions, and infected equids may act as carriers for several years (as in *T. equi* infection) or for the rest of their lives, thereby acting as reservoirs (3, 11–13).

For the diagnosis of EP, light microscopy is used to identify intraerythrocytic parasites (piroplasm stage) in thin blood smears prepared from acutely infected animals; however, in case of chronic or inapparent infections, parasitaemia is too low to be detected using thin blood smears (3). To overcome such problems, several serologic tests have been developed; including the complement fixation test (CFT), the indirect fluorescent antibody test (IFAT), and the enzyme-linked immunosorbent assays (ELISAs) mostly used for large-scale epidemiological studies (3, 5, 6, 14, 15). The competitive ELISAs (cELISA) employing monoclonal antibodies to recombinant antigens of *T. equi* (merozoites antigen 1; EMA-1) and *B. caballi* (rophtry associated protein; RAP-1) have been reported to be successful in detection of antibodies in sera of horses from different parts of the world (16, 17) and cELISA is one of OIE prescribed tests for International Trade (18). In addition, molecular methods (such as polymerase chain reaction-based tools) to detect and quantitate parasite DNA in host blood have also been developed and are considered reliable diagnostic tools (19–21).

In Pakistan, our knowledge on the prevalence and distribution of EP in different equids (horse, donkeys and mules) is very limited (22, 23, 16) and most of these studies, except (16), were based on conventional blood smear examination of *T. equi* and *B. caballi* which is less sensitive method and can give false negative results. Recently, cELISAs was used to determine the seroprevalence of *B. caballi* and *T. equi* in donkey, horses, and mules in five major metropolises of Punjab province and found that 52.6% (226/430) equids were seropositive for EP (16). We extended our studies on EP in other parts of Pakistan and the present project was as designed to determine the seroprevalence and risk factors associated with *T. equi* employing cELISA in two equine populated districts (Peshawar and Charsadda) of Khyber Pakhtunkhwa (KPK) province of Pakistan.

## Materials and Methods

### Study areas and meteorological characteristics

**Fig.1:**
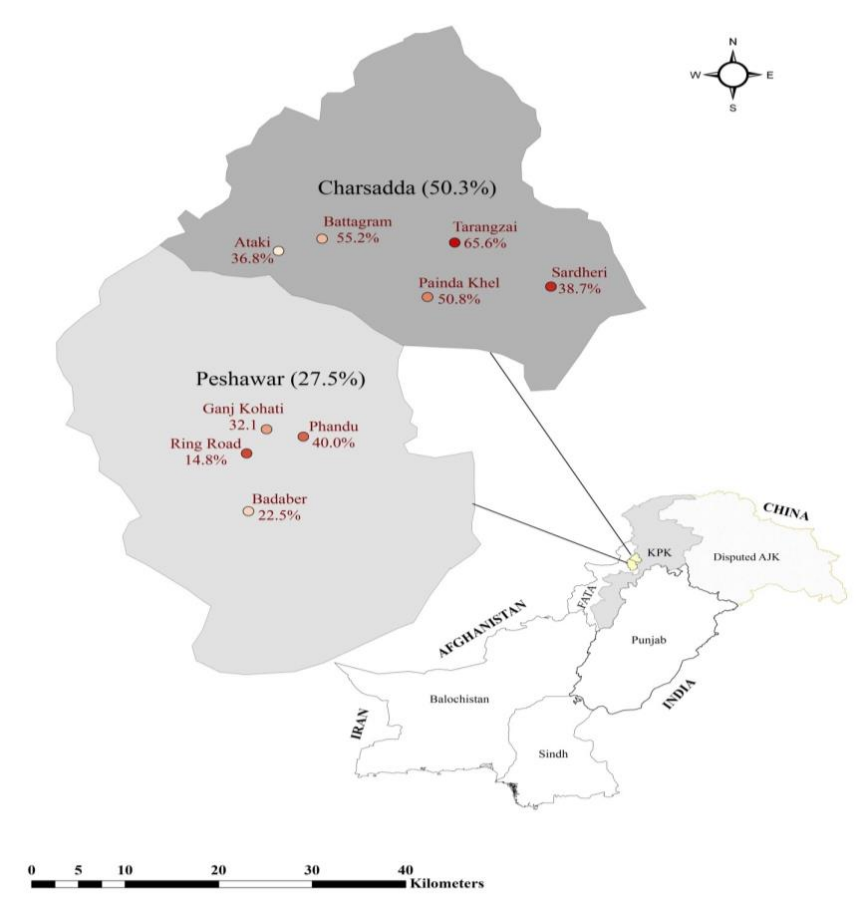
Choropleth map showing prevalence of *Theileria equi* in different areas of Khyber Pakhtunkhwa Province, Pakistan

A cross-sectional sero-epidemiological survey was conducted to estimate the antibodies against *T. equi* in three species of equids (donkey, horse and mule) from Sep 2012 to Mar 2013 in two draught equine populated districts of Peshawar (34°01′N 71°35′E) and Charsadda (34°09′N 71°44′E) in Khyber Pakhtoonkhawa (KPK) Province of Pakistan (Fig. 1). Peshawar has a semi-arid climate with very hot summers (25–40 °C), mild winters (4–18 °C) and an average annual precipitation of 384 mm, whereas Charsadda has mild summer (21–30 °C) and winter (6–19 °C) with an average annual precipitation of 460 mm. The selection of these districts was based on the presence of a maximum number of working equines in KPK (24).

### Demography and husbandry practices of the study population

According to the livestock census of Pakistan (24), Peshawar has a population of 18358 donkeys, 2368 horses, and 421 mules, whereas Charsadda has 35262, 6205 and 473 donkeys, horses and mules, respectively. Owing to the low socioeconomic condition of the owners, in both districts, draught equines generally have poor body condition due to heavy workload and poor level of nutrition. Alfalfa is the predominant fodder fed and is supplemented with chickpea. No immunization and mineral–vitamins supplementation program is in place.

### Sampling frame

As the prevalence of EP is unknown in KPK, the sample size was calculated by considering the expected prevalence to be 50% with confidence limits of 95% and a desired absolute precision of 5% to collect a maximum number of samples. The number of samples thus calculated was adjusted for finite population (25) and a total of 393 equines (195 horses, 194 donkeys, and 4 mules) serum samples were collected from five and four randomly selected localities in Charsadda (total number of samples=193) and Peshawar (n=200), respectively (Table 1). Based on age, the equids were categorized into two groups: Group 1 comprised of ≤10 yr-old animals; whereas Group 2 included > 10 yrold equines.

**Table 1:** Seroprevalence of *Theileria equi* in equines in different localities of two districts (Charsadda and Peshawar) from Khyber Pakhtunkhwa province, Pakistan

***District***	***Locality (n)***	***Seroprevalence % (proportion)***	***95% C.I.***	***P-value***
Charsadda	Ataki (38)	36.8 (14/38)	21.8–54.0	Χ^2^ = 7.63
	Batagram (29)	55.2 (16/29)	35.7–73.6	*P* = 0.106
	Penda Khel (63)	50.8 (32/63)	37.9–63.6	
	Sarderi (31)	38.7 (12/31)	21.8–57.8	
	Torenzai (32)	65.6 (21/32)	46.8–81.4	
Peshawar	Ganjkohati (56)	32.1 (18/56)	20.3–46.0	χ^2^ = 9.39
	Badabir (40)	22.5 (9/40)	10.8–38.5	*P* = 0.024
	Pandu (50)	40.0 (20/50)	26.4–54.8	
	Ring Road (54)	14.8 (8/54)	6.6–27.1	
Total		38.2 (150/393)	33.3–43.2	

n: number of animals

### Sample collection and animal data recording

The present study was conducted after the approval of Research Synopsis Scrutiny Committee, Faculty of Veterinary Science, University of Agriculture, Faisalabad, Pakistan.

Blood samples (∼ 5 mL) were drawn into anticoagulant coated and free vacutainers (Improvacuter, Hamburg, Germany) and labeled accordingly. Following centrifugation (4000 rpm, 10 min), sera were harvested and preserved (−20 °C) until tested. Complete blood count was estimated by hematology analyzer (Merck Diagnostic, Germany). In addition, a questionnaire was also designed in order to investigate the risk factors associated with EP and the data characteristics of the sampled animals (species, gender, age, and location), and the history, management and treatment of EP were obtained by interviewing the owners. Complete physical examination of animal sampled was performed and values regarding vital physiological parameters and observations were recorded.

### Serological examination (cELISA)

All serum samples were tested using a commercially available cELISA kit (VMRD, Inc., Pullman, USA) for *T. equi* according to manufacturer’s instructions. This assay detects serum antibodies against recombinant *T. equi*merozoites antigen 1 (EMA-1), a surface protein on merozoites of *T. equi* (17). The optical density (O.D.) was measured using an ELISA plate reader and percent inhibition was calculated using the following formula mentioned by the manufacturer:

Percent Inhibition (% I): = 100 - [(Sample O.D. × 100) ÷ (Mean Negative Control O.D.)] All samples producing ≥ 40% inhibition were regarded as positive whereas those with values <40% were considered negative.

### Statistical analyses

Data collected were categorized and prevalence along with 95% confidence interval (CI) was calculated as previously described (16)*.* Chi-square (χ^2^) test was performed to measure the significance (*P*<0.0) of association between different variables (district, age, sex, species, presence of ticks, history of voiding red urine, anaemia and seroprevalence). Bivariable analysis was conducted and odds ratio (OR) along with 95% CI was calculated for each variable. Finally, a multivariate logistic regression analysis was conducted to assess the association between seroprevalence of *T. equi* and variables found significant in the initial bivariable screening (16). Data were analyzed using IBM SPSS Statistics 17.0 for Windows® (IBM Corporation, Route 100 Somers, New York, USA).

## Results

Overall, 150 (out of 393; 38.2%, 95% CI=33.3–43.2) equids were tested positive for antibodies against *T. equi*. Seroprevalence of *T. equi* was significantly different (*P*<0.001) between Charsadda (50.3%) and Peshawar (27.5%), and it varied from 36.8% to 65.6% in five localities in Char-sadda (*P*=0.106) and 14.8% to 40.0% in 4 locations of Peshawar (*P*=0.024) (Fig. 1; Table 1).

Since no mule was found positive for the antibodies against *T. equi*, they were excluded from the analysis as found statistically insignificant. Higher seroprevalence was recorded in donkeys (50.5%) than horses (26.7%; χ^2^=23.35, *P*<0.001), in ≤10 yr-old (50.6%) than 10 yr-old animals (29.4%; χ^2^=18.08, *P*<0.001); in females (39.8%) than males (35.9%; χ^2^=0.55, *P*=0.45), and in tick-infested equines (65.8%) than tick-free subjects (35.6%; χ^2^=13.18, *P*<0.001). Anemia was recorded in 46.1% of *T. equi* positive equines while 29.8% of seropositive equines did not exhibit signs of anemia (χ^2^=10.88, *P*=0.001). Similarly, equines having history of voiding red urine had a higher (*P*<0.001) seroprevalence (67.6%) than those showing no such clinical sign (35.8%).

Univariable analysis of localities indicated that equines belonging to the district Char-sadda had a higher likelihood (OR 2.66, 95% CI 1.75–4.06) to be test-positive for antibodies against *T. equi* (Table 2). Similarly, analyses of other variables revealed that donkeys (OR 2.81, 95% CI 1.84–4.29), ≤ 10 yr-old equines (OR 2.46, 95% CI 1.62–3.74), females (OR 1.18, 95% CI 0.76–1.83), tick-infested equines (OR 3.48, 95% CI 1.72–7.04), and equines with history of red urine (OR 3.75, 95% CI 1.77–7.95) and anemia (OR 2.02, 95% CI 1.33–3.07) were more likely to be positive for *T. equi* (Table 2).

**Table 2:** Risk factors for the seroprevalence of *Theileria equi* in horses (n=195) and donkeys (n=194) from two districts (Charsadda and Peshawar) of Khyber Pakhtunkhwa Province, Pakistan

***Variable Name***	***Category***	***Seroprev. % proportion)***	***95% CI***	***OR***	***95% CI***	***P value***
District	Charsadda (189)	50.3 (95/189)	(42.9–57.6)	2.66	1.75–4.06	χ^2^ = 21.25; *P*< 0.001
	Peshawar (200)	27.5 (55/200)	(21.4–34.2)	1.00	-	
Host species	Donkey (194)	50.5 (98/194)	(43.3–57.8)	2.81	1.84–4.29	χ^2^ = 23.35; *P*< 0.001
	Horse (195)	26.7 (52/195)	(20.6–33.5)	1.00	-	
Age	≤ 10 yr (168)	50.6 (85/168)	(42.8–58.4)	2.46	1.62–3.74	χ^2^ = 18.08; *P*< 0.001
	> 10 yr (221)	29.4 (65/221)	(23.5–35.9)	1.00	-	
Sex	Female (261)	39.8 (104/261)	(33.9–46.1)	1.18	0.76–1.83	χ^2^ = 0.55; *P* = 0.45
	Male (128)	35.9 (46/128)	(27.7–44.9)	1.00	-	
Presence of ticks	Yes (38)	65.8 (25/38)	(48.6–80.4)	3.48	1.72–7.04	χ^2^ = 13.18; *P*< 0.001
	No (351)	35.6 (125/351)	(30.6–40.9)	1.00	-	
History of red urine	Yes (34)	67.6 (23/34)	(49.5–82.6)	3.75	1.77–7.95	χ^2^ = 13.30; *P* < 0.001
	No (355)	35.8 (127/355)	(30.8–41)	1.00	-	
Anemia	Yes (208)	46.1 (96/208)	(39.2–53.2)	2.02	1.33–3.07	χ^2^ = 10.88; *P* = 0.001
	No (181)	29.8 (54/181)	(23.3–37.1)	1.00	-	

Four mules were excluded from the analysis, as they were found negative

All variables found significant in the bivariable analysis (*P*<0.2) were included in the final binary logistic regression analysis; however, sex and age were removed from the model at subsequent steps (*P*>0.05). Being a donkey (OR 2.94), having tick infestation (OR 4.32), history of voiding red urine (OR 3.97) and anaemia (OR 2.1) were the factors significantly associated with the seroprevalence of *T. equi* in sampled equines (Table 3).

**Table 3:** Multivariate logistic regression analyses for the prediction of *Theileria equi* in equines from two districts (Charsadda and Peshawar) of Khyber Pakhtunkhwa Province, Pakistan

***Exposure variable (n)***	***Comparison (n)***	***Odds Ratio***	***95% C.I.***		***P-value***
			***Lower***	***Upper***	
Donkey (194)	Horse (195)	2.94	1.874	4.608	0.000
Tick infested (38)	Tick free (351)	4.32	2.037	9.151	0.000
History of red urine (34)	No history (355)	3.97	1.790	8.785	0.001
Anaemic (208)	Normal (181)	2.10	1.311	3.246	0.002

n: number of animals

## Discussion

This study provides the first insights into the seroprevalence of EP (caused by *T. equi*) in donkeys, horses and mules in the two most equine populated districts (i.e., Peshawar and Charsadda) of KPK, employing cELISA - an OIE recommended serological test. Previously, a number of studies used a less sensitive technique, the demonstration of piroplasms on Giemsa stained blood smears for the diagnosis of *B. caballi*and *T. equi* in Pakistan (22, 23, 26); however, this technique is rarely conclusive and chances of false negative results are usually high, particularly when parasitaemia is low. *T. equi* infection is believed to be more prevalent and widespread than that of *B. caballi* (5) and carrier animals are significantly important globally because of the risk of spreading infection into a non-endemic region. Therefore, it is crucial to utilize sensitive diagnostic assays in epidemiologic studies and to identify the carriers of EP. Previously, first time cELISA was used to determine the seroprevalence of EP in equines in Punjab Province of Pakistan and herein the same serological assay was used to determine the seroprevalence of *T. equi* in KPK, a northwest province of Pakistan (16).

We found that the overall seroprevalence of *T. equi* was 38.2%. A wide range was reported (2.6%–83.5%) of seroreactivity against *T. equi*in equines (16, 27–32). However, the comparison between the results of different studies should be interpreted with extreme caution as differences in climatic conditions, number of samples tested, sensitivity and specificity of employed diagnostic tests and the presence of competent vectors can affect the prevalence of EP in equines (6, 33, 34).

Seroprevalence of *T. equi* infection was significantly higher (*P*<0.001) in Charsadda district (50.3%) than Peshawar district (27.5%). This difference could be attributed to differences in husbandry, management, tick control measures, activity of equids and the presence of more number of carrier animals; and such factors have been found as potential risk factors in previous studies (10, 16, 34). Moreover, the transmission of *T. equi* among equines is influenced by the spatial distribution and dynamics of competent vector (ticks) populations. As the vector populations are dependent upon prevailing climatic conditions (35, 36), the climatic conditions are more suitable for the propagation of ticks in Charsadda than Peshawar as an average annual precipitation rate in the former district is 460 mm as compared 384 mm in Peshawar, thus contributing to higher prevalence of *T. equi* in Charsadda. However, this proposal requires further testing as our knowledge on bionomics of ticks in these districts is very limited.

In the present study, the presence of ticks had a positive association (*P*<0.001) with the seroprevalence of *T. equi*. This finding is in agreement with those of previous studies documenting an increased incidence of *T. equi* in tick-infested equids (12, 37, 38). In this study, attempt was not made to identify ticks, which could potentially be responsible for the transmission of *T. equi*. Although a previous study (39) on the prevalence of ticks infesting equines in these two regions found the presence of four genera of ixodid ticks (*Amblyomma*, *Boophilus, Hyalomma* and *Rhipicephalus*) known to be vectors of *T. equi* (40, 3, 41, 4).

Seroprevalence of *T. equi* was higher (*P*<0.001) in ≤ 10 yr-old (50.6%) than > 10 yr-old (29.4%) animals. This difference in *T. equi* antibodies in two age groups is not concordant with previous findings that documented that the seroprevalence of EP increased with an increase in age of equines (41). This discrepancy should be interpreted carefully as in the present project, equines were classified into two age groups i.e., ≤10 and >10 yr-old animals whereas (41) divided animals into three categories such as young, adult and geriatric and the number of animals in the latter category was small.

Seroprevalence of *T. equi* infection in donkeys (50.5%) was significantly higher (*P*<0.001) than horses (26.7%). This finding is contrary to that of a previous study (16) that conducted their study in five metropolises of a neighboring province (Punjab) of KPK in Pakistan and found that seroprevalence of *T. equi* was significantly higher in horses than donkeys and mules. This discrepancy in seropositivity in two different provinces could be attributed to differences in husbandry and parasitic control practices as well as demography. In both districts of Charsadda and Peshawar, donkeys are raised in rural areas under poorer management conditions and the parasitic control programs are rarely practiced. Donkeys are usually more exposed to outdoor activities like grazing as compared to other equids, which is also a potential risk factor for the transmission of *T. equi* via ticks (37, 42). In addition, chronic cases of EP are more common in the donkeys as compared to other equines and most of the affected animals remain seroreactive throughout their life (43).

Anemia is a characteristic feature of *T. equi* infection (16). In the present study, a relatively high and statistically significant (*P*<0.001) seropositivity of *T. equi* was observed in anaemic (46.1%) as compared to non-anemic (29.8%) equines. Similarly, equines with a history of voiding red urine had higher (67.6%) and statistically significant (*P*<0.001) seroprevalence of *T. equi* than those without it (35.8%). These findings are consistent with the results of previous studies conducted on *T. equi* (5, 10, 11)

## Conclusion

Female animals had slightly higher risk of being seropositive (39.8%) as compared to males (35.9%), although the results were non-significant (*P*=0.45). This negligible gender predisposition is a confounding factor and might be attributed to higher number of female samples tested herein. However, an increased susceptibility of female animals to protozoan infections (44) has been attributed to immunosuppression as a result of environmental, nutritional, lactation and pregnancy stresses (45, 46) in dairy animals.

EP caused by *T. equi* is endemic donkeys and horses in the KPK Province of Pakistan. Future studies should focus to understand the epidemiology of disease and devise control measures. Conventional and molecular techniques should be used to identify the competent tick vectors along with seasonal distribution and other factors responsible for the spread and maintenance of EP in KPK as well as other equine populated regions of Pakistan.
